# Video Classification of Cloth Simulations: Deep Learning and Position-Based Dynamics for Stiffness Prediction

**DOI:** 10.3390/s24020549

**Published:** 2024-01-15

**Authors:** Makara Mao, Hongly Va, Min Hong

**Affiliations:** 1 Department of Software Convergence, Soonchunhyang University, Asan 31538, Republic of Korea; makaramao07@gmail.com (M.M.); vahonglykhmer@gmail.com (H.V.); 2 Department of Computer Software Engineering, Soonchunhyang University, Asan 31538, Republic of Korea

**Keywords:** cloth simulation, position-based dynamics, multi-label, Transformer, video classification, deep learning

## Abstract

In virtual reality, augmented reality, or animation, the goal is to represent the movement of deformable objects in the real world as similar as possible in the virtual world. Therefore, this paper proposed a method to automatically extract cloth stiffness values from video scenes, and then they are applied as material properties for virtual cloth simulation. We propose the use of deep learning (DL) models to tackle this issue. The Transformer model, in combination with pre-trained architectures like DenseNet121, ResNet50, VGG16, and VGG19, stands as a leading choice for video classification tasks. Position-Based Dynamics (PBD) is a computational framework widely used in computer graphics and physics-based simulations for deformable entities, notably cloth. It provides an inherently stable and efficient way to replicate complex dynamic behaviors, such as folding, stretching, and collision interactions. Our proposed model characterizes virtual cloth based on softness-to-stiffness labels and accurately categorizes videos using this labeling. The cloth movement dataset utilized in this research is derived from a meticulously designed stiffness-oriented cloth simulation. Our experimental assessment encompasses an extensive dataset of 3840 videos, contributing to a multi-label video classification dataset. Our results demonstrate that our proposed model achieves an impressive average accuracy of 99.50%. These accuracies significantly outperform alternative models such as RNN, GRU, LSTM, and Transformer.

## 1. Introduction

Cloth simulation in computer graphics endeavors to replicate the dynamic behavior of fabrics within a virtual environment [1]. This involves representing cloth as a mesh of vertices and faces with assigned physical properties such as mass and stiffness [2]. Forces like gravity and wind, coupled with constraints for stretching, bending, and collision avoidance, shape the movement of cloth [3]. The mesh evolves through iterative integration, giving rise to lifelike cloth interactions, which are rendered with lighting, shading, and textures [4]. Despite its computational complexity, cloth simulation is vital for realism in gaming, animation, and virtual fashion design applications [5].

In video classification, Deep Learning has emerged as a powerful tool, offering researchers avenues for exploration in various domains such as object prediction, recommendation systems, natural language processing, et al. [6,7,8,9]. However, video classification using Deep Learning (DL) techniques presents unique challenges, including spatial-temporal feature extraction, handling large-scale and diverse datasets, real-time processing constraints, and model interpretability. Innovations in recurrent neural networks (RNNs), gated recurrent units (GRUs), long short-term memory (LSTM), Transformer, and other models are overcoming these challenges. As mentioned in the introduction, we will provide detailed presentations on the advantages and disadvantages of our models in the experiment results section. This analysis will judiciously guide our exploration into the problem addressed in this paper, ensuring a comprehensive understanding of the intricacies involved in overcoming these challenges. Innovations in RNNs, GRUs, LSTM, Transformer, and other techniques will be scrutinized to showcase their effectiveness in addressing the unique complexities of video classification [10,11].

Position-Based Dynamics (PBD) cloth simulation is pervasive in various industries, notably animation, faithfully emulating the behavior of virtual cloth to mirror real-world characteristics. This versatile approach in computer graphics for simulating cloth and soft-body dynamics represents cloth as interconnected particles governed by constraints. This methodology allows for the modeling of physical properties such as position, velocity, and mass. The advantage of PBD lies in its capacity to maintain structural integrity and dynamics through constraint-based methods, employing distance, angle, and position constraints to enforce particle relationships. Additionally, PBD relies on iterative constraint solving, facilitating real-time performance and effective collision handling. However, the adjustment of virtual cloth behavior by modulating stiffness introduces a persistent complexity in predicting and classifying physical properties derived from visual cloth simulations. Despite this challenge, PBD remains a powerful tool due to its stability, realism in cloth motion, and efficient real-time performance [12].

This paper introduces a proposition of DL techniques to enhance predictions in the context of multi-label video classification [13]. The dataset used in this study, comprising over 3600 videos, was created through the PBD method for cloth simulation. These videos were utilized in experiments related to multi-label video classification [14]. Throughout this work, ImageNet models (DenseNet121, ResNet50, VGG16, and VGG19) models operate by extracting additional frame-to-frame information from the videos [15,16,17], with the primary objective of facilitating predictions within the context of multi-label video classification. However, it is noteworthy that the performance of convolutional neural networks (CNNs) remains suboptimal when dealing with video datasets featuring consistent objects across different videos [18]. The results demonstrate a superior performance of the Transformer model compared to the other approaches.

Recent research studies have introduced promising methodologies with significant advancements. In the work by Rafiq et al. [19], they propose the utilization of DL techniques to predict video classifications, employing a pre-trained AlexNet CNN for scene analysis. Their innovative approach incorporates fully connected layers in an encoder architecture. Meanwhile, Rashidi Fathabadi et al. [20] focus on enhancing laparoscopic surgery skills through simulation experiences within the Fundamentals of Laparoscopic Surgery (FLS) training. They employ an intelligent box-trainer system equipped with cameras and fuzzy logic assessment for skill development. Notably, their study involves a group of physicians and provides autonomous assessment results shortly after the training, demonstrating an efficient and cost-effective means of improving laparoscopic surgical proficiency.

In another contribution, Zhang et al. [21] underscore the pivotal role of DL techniques across diverse domains, emphasizing the continuous evolution of DL models and methods. Furthermore, they announce the forthcoming publication of a special issue containing fourteen research papers dedicated to deep-learning-based sensing, imaging, and video processing, marking a significant stride in the field’s progress. Malm et al. [22] focus on developing and testing conductive coatings for textiles, considering factors such as electrical conductivity, fabric stiffness, and durability, particularly for their application in electronic textiles (e-textiles). Their research highlights the potential revolutionary impact of fusing computer simulation, specifically using the Position-Based Dynamics (PBD) method for generating video cloth datasets with deep learning. This approach aims to significantly enhance realism and accuracy in cloth simulation and other computer graphics applications, representing real-world materials and behaviors. The study’s key contributions are as follows:

- Generating an exclusive collection of cloth video simulations using the PBD method consisting of 1280 videos per category. The dataset itself is extensive, featuring approximately 3840 videos distributed across three categories. This diversity enables experimentation across five distinct classes;
- The construction of four foundational models from scratch, including RNN, GRU, LSTM, and Transformer models, to classify the five cloth simulation dataset classes;
- The utilization of pre-train models with parallel processing for GPU-based fine-tuning, including the ImageNet (DenseNet121, ResNet50, VGG16, and VGG19)—the highest-performing pre-trained model—integrated with custom CNNs and dense layers;
- Comparison of CPU and GPU processing for pre-training during video frame analysis.

The rest of this paper is organized as follows: Section 2 covers the related work in the video classification. The proposed method is presented in Section 3, the experiment results, discussion, and comparison are in Section 4, and finally, Section 5 concludes the work and suggests future aspects.

## 2. Related Works

Many of the existing approaches propose DL techniques for image or video classification. Mao et al. [23] presented significant work on using a mass-spring system (MSS) for cloth simulation to generate a dataset for video classification. They applied random rotation techniques to the pre-trained dataset using CNN models and achieved a classification accuracy of 99.01% on five categories of video classification. On the other hand, Vijeta et al. [24] conducted a comprehensive systematic literature review (SLR) in video processing using DL. Their study delves into the applications, functionalities, techniques, datasets, issues, and challenges with 93 research articles from reputable databases spanning 2011 to 2020. This SLR serves as a valuable resource, guiding researchers through recent literature, available datasets, and established DL methodologies for video processing.

Additionally, Wu et al. [25] proposed an innovative hybrid DL framework for video classification. Their approach effectively captures static spatial information and short-term temporal cues in videos by employing two separate CNNs. These CNN-based features are subsequently fused within a regularized feature fusion network, enabling the model to learn and exploit feature relationships for improved performance. Notably, their method achieved an archive-high accuracy of 91.3% on the UCF-101 dataset and 83.5% on the CCV dataset.

Karpathy et al. [26] conducted a comprehensive study using CNN for large-scale video categorization. They tested various techniques to improve temporal connectivity and introduced a multiresolution fused architecture to speed up training. Their spatiotemporal networks showed a modest improvement over single-frame models (59.3% to 60.9%) and significant gains over feature-based baselines (55.3% to 63.9%). Retraining on the UCF-101 dataset further enhanced model performance compared to the baseline.

Jiang et al. [27] developed a hybrid deep learning framework that combines multiple modalities (appearance, motion, and audio) to enhance video classification. They used CNN for feature extraction, fused features with a network, and employed LSTM networks for long-term temporal dynamics. Their framework improved video classification by leveraging various multimodal features.

Yue-Hei Ng et al. [28] introduced novel deep neural network architectures to integrate image information over longer video sequences efficiently. They proposed two methods for handling full-length videos. The first method explored various convolutional temporal feature pooling designs to adapt CNN for this task. The second method used LSTM cells to model videos as ordered sequences of frames connected to the CNN’s output. Their top-performing networks significantly outperformed previous results on the Sports 1 million dataset (73.1% vs. 60.9%) and the UCF-101 datasets, both with (88.2% vs. 87.9%) and without additional optical flow information (82.6% vs. 72.8%).

## 3. Methods and Methodology

### 3.1. Pre-Train Models

In this section, we introduce a Convolutional Neural Network (CNN) designed for clothing category classification, utilizing an extensive clothing category dataset. We propose the use of various neural architectures on our pre-trained model, including DenseNet121, which requires a three-dimensional (3D) tensor input of size 128 × 128 × 3, as well as ResNet50, VGG16, and VGG19, all of which demand 3D tensor inputs sized at 224 × 224 × 3 for color image processing. Detailed descriptions of our pre-trained models are provided in Table 1.

**Figure 1:**
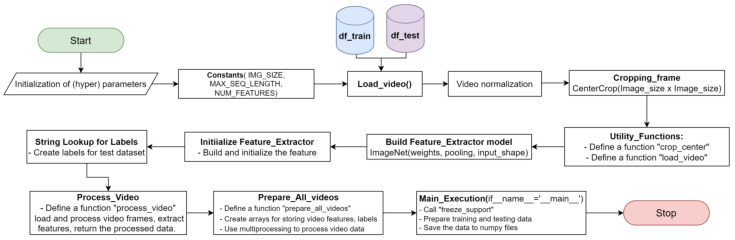
Parallel Processing Flowchart for Reading Video Frames.

In our data processing pipeline, as depicted in Figure 1, we incorporate parallel processing techniques to enhance video frame retrieval efficiency. The initial steps encompass library imports and the definition of critical constants of parameters such as image dimensions, sequence length, and feature extraction. Data loading operations involve the extraction of training and testing data from CSV files, including frame cropping and video data retrieval. This process employs a method that alternates between reading and skipping two frames, culminating in processing the entire 960-frame video. This methodology is designed to optimize computational resources and enhance parallel processing capabilities. The key strategy involves selectively reading and skipping frames in a staggered pattern, resulting in a substantial improvement in computational efficiency. By strategically choosing frames, we strike a balance between maintaining relevant information and mitigating the computational burden, ultimately saving both computing resources and associated costs. This selective approach is particularly advantageous in scenarios requiring real-time analysis, where the reduction in overall processing time is crucial. The methodology ensures that only pertinent frames are processed, contributing to an efficient and cost-effective video analysis system.

This approach optimizes both processing time and the utilization of parallel processing capabilities. Furthermore, we construct a feature extractor model based on the architectural insights from the ImageNet models (DenseNet121, ResNet50, VGG16, and VGG19), complemented by creating a label vocabulary for the test dataset. In our video processing pipeline, we employ specialized functions for frame processing and feature extraction, utilizing multiprocessing techniques. During frame processing, each frame undergoes specific operations, including a random rotation of up to 70 degrees and a random shift of 224 × 224 pixels. Simultaneously, feature extraction is conducted in parallel on multiple frames, harnessing multiprocessing to improve efficiency. This methodology is crafted to optimize computational resources and minimize processing time. Lastly, the main execution section, triggered by the ‘if __name__ == ‘__main__’,’ condition, encompasses data preparation for both training and testing, with dimensions clearly defined and the processed data saved as NumPy files.

**Figure 2:**
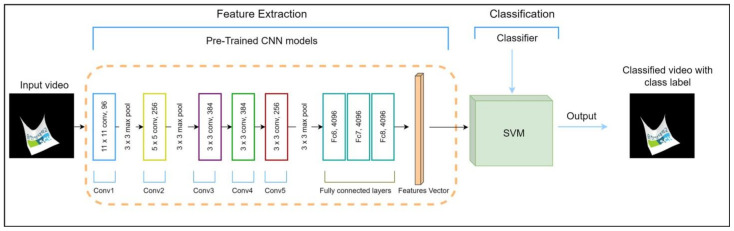
Pre-train using CNN models: DenseNet121, ResNet50, VGG16, and VGG19.

To enhance the effectiveness of our approach, we systematically investigate a variety of deep learning models, including recurrent neural networks (RNN), gated recurrent units (GRU), long short-term memory (LSTM), and the Transformer architecture. Additionally, we extend our analysis to encompass pre-trained CNN models obtained from ImageNet, specifically DenseNet121, ResNet50, VGG16, and VGG19, as depicted in Figure 2. These pre-trained models come equipped with extensive feature representations derived from a vast video dataset, capitalizing on the advantages of transfer learning embedded within these well-established architectures.

**Figure 3:**
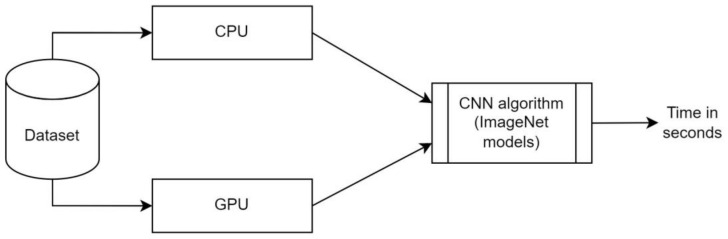
System architecture of the comparative analysis of both CPU and GPU on a pre-trained process.

In this section, we introduce an integrated approach to efficiently process our dataset and execute the CNN algorithm, which is central to our research on video classification within cloth simulations, as shown in Figure 3. Our central processing unit (CPU) plays a pivotal role in various pipeline tasks, particularly during the pre-training stage, which involves training on 880 videos and testing on 400 videos. We specifically aim to compare the time it takes to read frames from videos when utilizing both CPU and graphics processing unit (GPU) resources. Tasks such as dataset management, preprocessing, and frame extraction are handled on the CPU, with optimized core allocation and parallel execution using pool techniques to expedite dataset preparation and reduce critical operation times.

Simultaneously, our GPU efficiently handles computational work, especially during CNN model training and inference. We use pool techniques to ensure optimal GPU core allocation and usage, enhancing frame-based video classification speed and performance. Time measurements are expressed in seconds and represented by digits, facilitating detailed comparisons. The results are presented in Table 2. Our approach uses pool techniques to harmonize CPU and GPU resources, achieving an efficient balance between frame reading and deep learning computations during the pre-training stage. This hybrid approach streamlines our research pipeline and maximizes overall system throughput, ultimately contributing to the success of our video classification model for cloth simulations.

### 3.2. Data Set

Position-Based Dynamics (PBD) is a versatile approach in computer graphics for simulating cloth and soft-body dynamics. It represents cloth as interconnected particles governed by constraints, allowing for modeling physical properties such as position, velocity, and mass. PBD relies on constraint-based methods to maintain structural integrity and dynamics, employing distance, angle, and position constraints to enforce particle relationships. Through integration schemes and physics-based principles like Newton’s laws, PBD updates the particle positions to satisfy the constraint condition, ensuring stable and realistic cloth motion. The core of PBD lies in iterative constraint solving, enabling real-time performance and collision handling. However, applications of PBD go beyond cloth simulation. Its adaptability extends to various soft-body simulations, providing artists with the necessary control to achieve specific visual effects and behaviors. Details regarding the parameters used to generate the cloth dataset are shown in Table 3.

In addition to the constant parameters employed in this experimental dataset, several key variables have been utilized to shape the behavior of the simulations. The ‘Duration’ parameter, set to 30 s, defines the temporal extent of the cloth simulation, influencing the duration of the video simulation run. The ‘Mass,’ which ranges from 10 to 40, signifies the mass of individual particles or nodes within the cloth. It significantly impacts how the material reacts to external forces such as gravity and external interactions. Higher mass values impart greater resistance to deformation, while lower values promote enhanced flexibility and responsiveness.

The ‘Damping’, varying from 0.001 to 0.01, governs the rate at which motion or oscillation diminishes during the simulation. Higher damping values expedite motion damping, mimicking behavior in denser mediums, whereas lower damping values sustain motion and oscillations. The ‘Dt’ parameter, representing the time step, ranges from 0.01 to 0.05 and determines how frequently the simulation updates. Choosing smaller time steps increases precision but requires more computational resources and may lead to slower simulations. On the other hand, larger time steps are computationally efficient but can introduce instability.

The ‘Iteration’ parameter ranges from 5 to 50 and represents the number of iterations in the cloth simulation. Increasing the iteration value results in a more detailed and refined simulation by allowing the cloth to undergo more iterations. However, this improvement comes at the expense of higher computational complexity.

**Figure 4:**
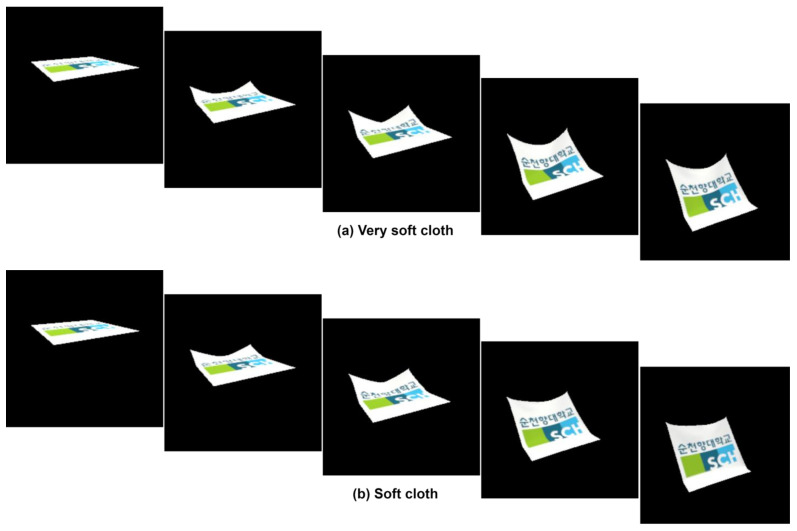

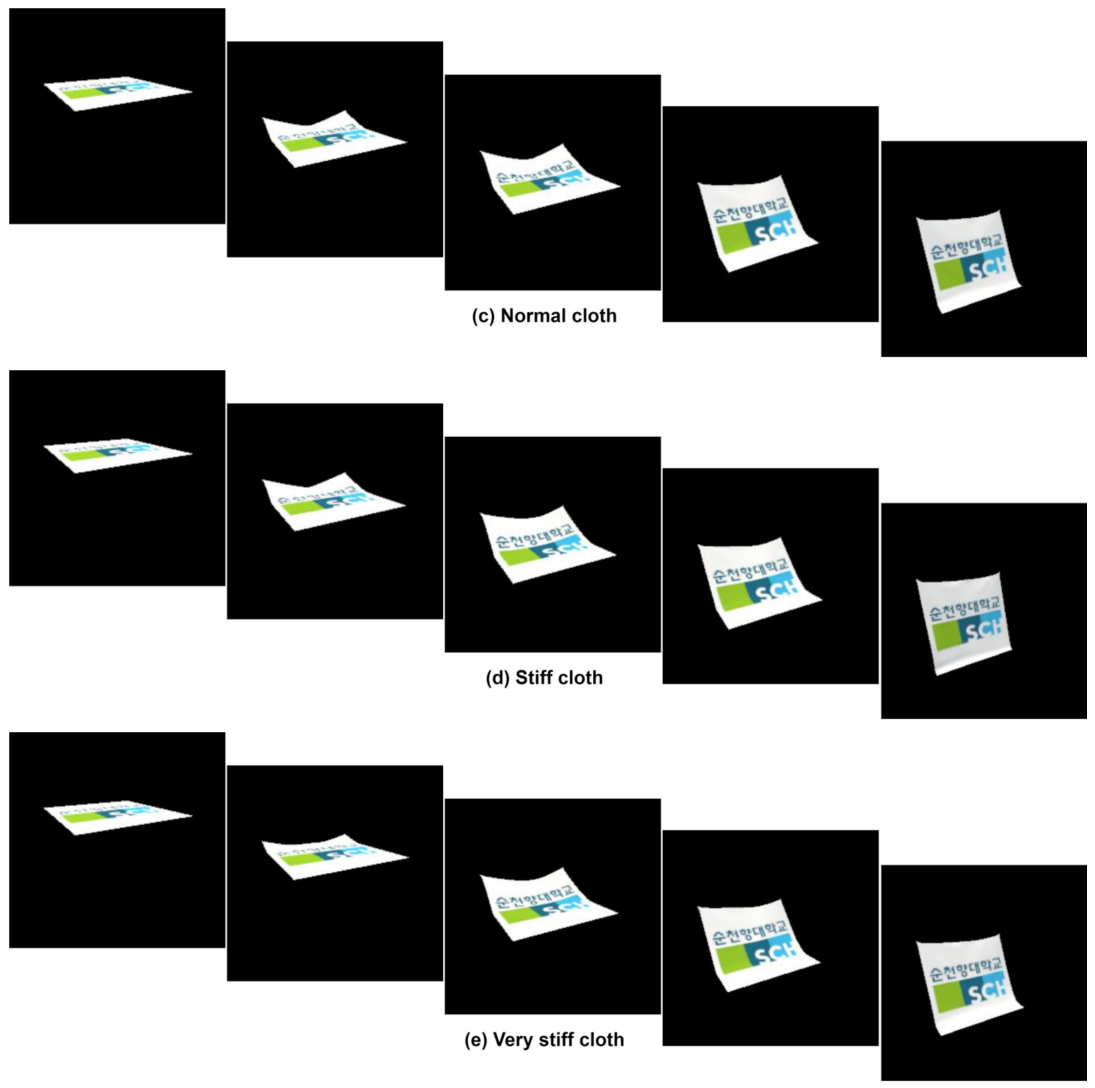
The video frames showcase various animations of flag waving in each category.

Lastly, ‘Stiffness’, commencing at 0.1 and reaching up to 1.0, governs the material’s rigidity or flexibility. Augmented stiffness values confer greater resistance to deformation, yielding a stiffer appearance, while diminished values foster enhanced flexibility and deformability within the material. In video dataset simulation, as shown in Figure 4, we defined a ‘stiffness’ scale ranging from 0.1 to 1.0, with 0.1 representing very soft cloth, 0.25 for soft cloth, 0.50 for normal cloth, 0.75 for stiff cloth, and 1.0 for very stiff cloth.

### 3.3. Data Augmentation

In this section, we introduce several techniques employed for our dataset, as utilized in the experiments presented in our paper. Since our video dataset frequently contains similar frames within individual videos, we have applied various strategies, including rotation, shifting, resizing, and flipping operations, to enhance accuracy during the pre-training of our video dataset.

**Figure 5:**
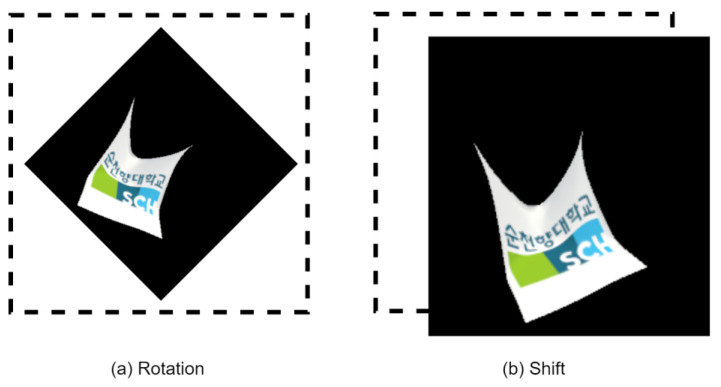
Data set augmentation.

We employ image rotation and shifting techniques to enhance the performance of our CNN models. Specifically, we apply a random rotation of up to 70 degrees to the video frames and perform a random shift of 224 × 224 pixels. These operations adjust the object’s orientation on the screen and ensure alignment with the target region for detection. This process is shown in Figure 5. Data augmentation is a fundamental step in improving the model’s performance and enhancing its ability to generalize from the input data, ultimately leading to increased accuracy.

This study investigates the classification of clothes generated from computer simulations, specifically focusing on cloth freefall, cloth collision, and cloth aerodynamics datasets. The PBD dataset classifies clothes of the same type with matching attributes. As shown in Table 4, each video is the same size (224 × 224), and all videos have a fixed length of 30 s, consisting of 960 frames. The nomenclature ‘PBD_Freefall_stf0.1_c0100.avi’ can be identified below: ‘PBD’ signifies the method used for generating the video dataset via computer simulation, ‘Freefall’ represents the type of animated objects that move in the video, ‘stf*.**’ conveys labels associated with the video categories, where the value ‘*.**’ is derived from computer simulations using the PBD method, and ‘c****.avi’ signifies the number of videos and type of videos.

**Figure 6:**
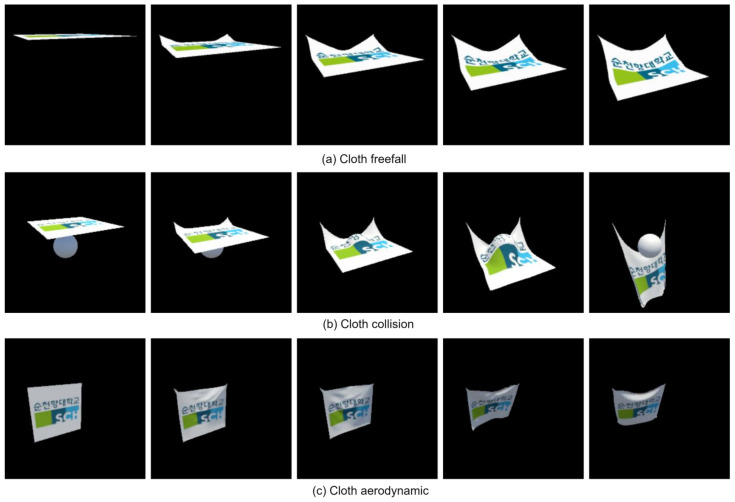
Shows animated videos depicting various movements captured from the video datasets. These movements include (**a**) cloth freefall, (**b**) cloth collision, and (**c**) cloth aerodynamics.

In Figure 6, the depicted object was generated through computer simulation employing the PBD method as part of our experimental process, encompassing the simulation of cloth freefall, cloth collision, and cloth aerodynamics.

### 3.4. Overview of Models

**Figure 7:**
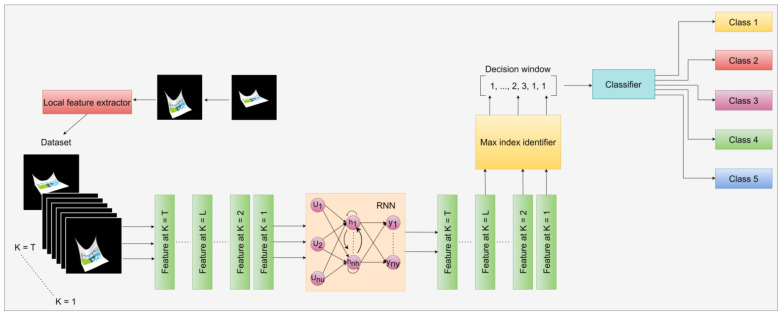
The architecture of the RNN model overview process flow works on video classification.

In this section, we provide an overview of the methods employed in this paper and present the corresponding results. We present an innovative approach for comparing an existing model with results from prior experiments is innovative. The recurrent neural network (RNN) model is a DL architecture commonly employed for video classification tasks, specifically designed to capture temporal dependencies and patterns in sequential data. A key component of RNNs is the hidden state, which functions as a memory element retaining information from previous inputs. As each frame is processed, the hidden state updates based on the current input and the preceding hidden state. This sequential updating mechanism enables the RNN to effectively capture temporal dependencies and gain insights into patterns over time. However, a challenge faced by traditional RNNs is the vanishing gradient problem, where gradients used for network training diminish over time, hindering the accurate capture of long-term dependencies. The RNN model can learn temporal patterns and dynamics essential for precise video classification by processing frames or patches individually. Through the analysis of sequential information, the RNN model can identify motion, detect temporal patterns, and generate predictions based on accumulated knowledge from previous frames. The process flow of the RNN model is illustrated in Figure 7.

**Figure 8:**
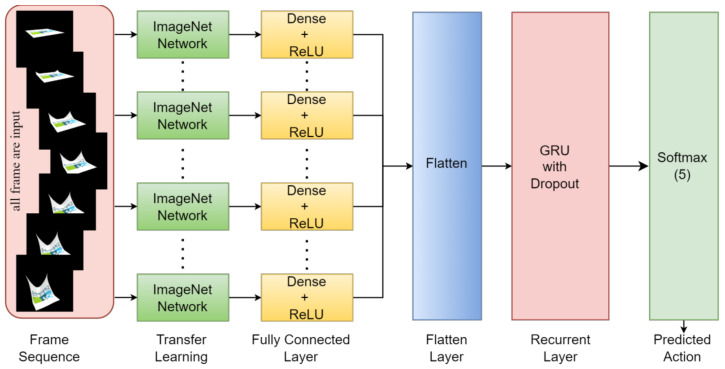
The architectural design with a recurrent full convolutional network and GRU layer for sequential frame processing leads to heatmap generation. Subsequent refinement and up-sampling achieve the desired spatial dimensions.

The gated recurrent unit (GRU), a recurrent neural network (RNN) family member, is commonly employed for image and video classification tasks. Originally developed as an extension of the conventional RNN, the GRU incorporates gating mechanisms that efficiently capture and propagate information within sequential data. In the context of image and video classification, the GRU model seamlessly processes frames or patches within images and videos sequentially. Its exceptional ability to capture long-term dependencies, efficient training, gradient stability, real-time processing capabilities, and adaptability to varying video lengths make it highly effective for video classification applications. Further details about the GRU model are shown in Figure 8.

**Figure 9:**
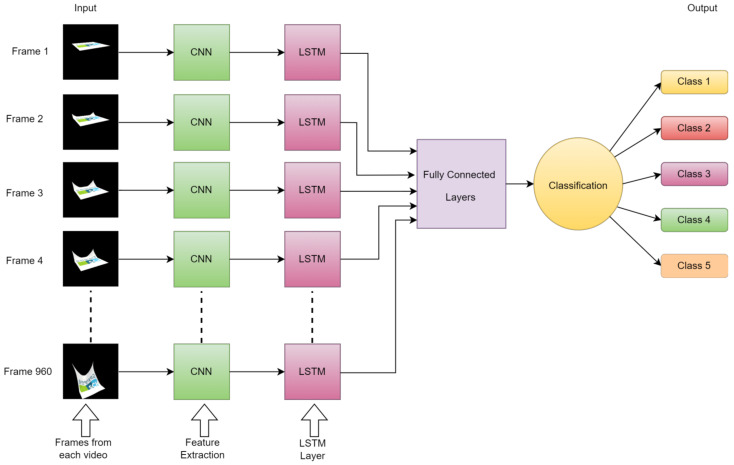
The architecture of the LSTM model overview process flow working on video classification.

As previously mentioned, LSTM models incorporate specialized memory cells that possess the ability to retain or discard information selectively as time progresses. This functionality is achieved through the utilization of three primary components: the input gate, responsible for determining the amount of new information to be stored in the memory cell; the forget gate, which controls the removal of past data from the cell; and the output gate, regulating the transfer of information from the memory cell to the subsequent time step. This gate-based mechanism empowers LSTMs to effectively capture and retain long-term dependencies while simultaneously addressing the challenge of the vanishing gradient problem. In video classification, the LSTM model can be sequentially applied to analyze the frames or segments of an image or video. By examining the temporal cues inherent in the video sequence, the LSTM model can learn and identify patterns, motion, and persistent temporal dynamics, as shown in Figure 9, which are crucial for achieving accurate video classification.

**Figure 10:**
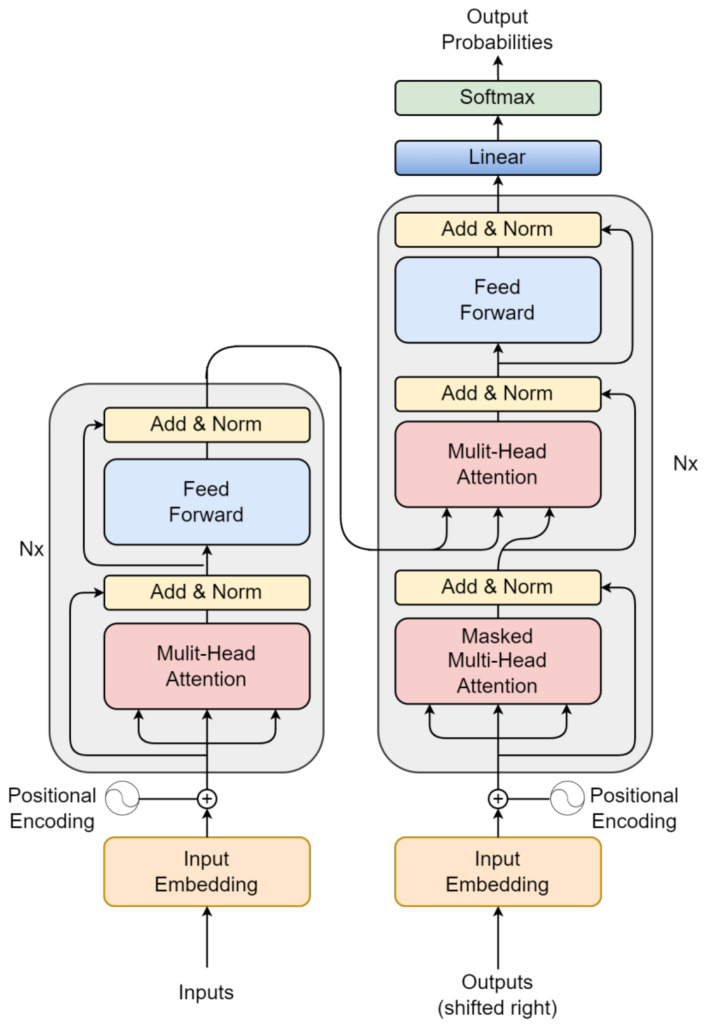
A transformer is typically constructed by stacking encoders linked together using provided input embeddings and output embeddings. This architecture relies exclusively on attention mechanisms in each encoder and decoder, eliminating the need for recurrent or convolutional operations.

The Transformer model for video classification, shown in Figure 10, represents a hybrid neural network architecture that merges the strengths of CNN and Transformer to excel at video recognition and categorizing videos. CNNs are adept at handling spatial features in image-related tasks, while transformers are skilled at modeling temporal dependencies and capturing extended context. The amalgamation of these two architectures results in a CNN-Transformer model that leverages both benefits, leading to superior performance in video classification. In this configuration, the CNN component is typically responsible for extracting visual features from each frame within the video, and these features are then passed into the Transformer component. The Transformer, in turn, models the temporal relationships between frames and generates the ultimate prediction for the video’s class label.

The Transformer model comprises multiple layers, and each layer is composed of two sub-layers: the self-attention mechanism and the feed-forward neural network. During forward propagation, the input data are processed through the self-attention sub-layer, which calculates attention weights between each input element and all others in the same layer. This enables the model to emphasize significant input elements while disregarding irrelevant ones. The output from the self-attention sub-layer is then transformed by the feed-forward neural network, applying nonlinear operations to the data and yielding a new representation.

In the backpropagation phase, the error signal travels backward through the network layers, commencing from the output layer and progressing toward the input layer. This error signal updates the parameters of the network, improving the object of model classification abilities. For object classification tasks, the Transformer model can be trained using labeled datasets, where each input is an image, and the corresponding output denotes the object’s class label within the image. Fine-tuning is often performed by optimizing a classification loss function employing techniques like stochastic gradient descent (SGD), Adam, or other optimization algorithms.

The Transformer model is a potent neural network architecture applicable to a broad spectrum of tasks, including object classification. Its effectiveness stems from its capability to efficiently capture extensive dependencies and create valuable representations of the input data.

### 3.5. Proposed Transformer Model Architecture

**Figure 11:**
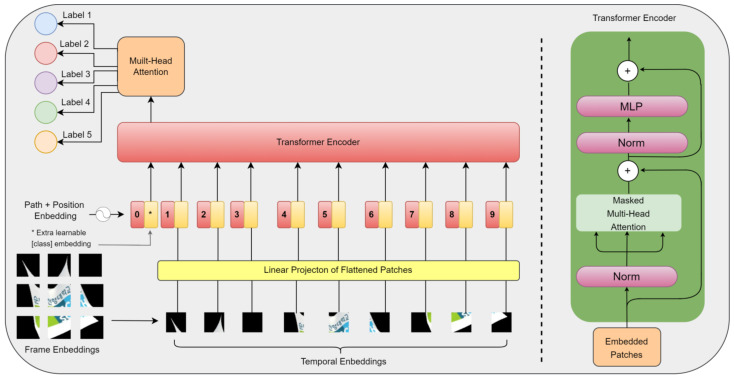
The process flow of the Transformer model for extracting frames as grids from a video, Including temporal embeddings.

Initially designed for natural language processing (NLP), the transformer model has applications in various domains, including video classification. The process flow for transformers working on video classification is depicted in Figure 11, and the details are provided below:

- Data Preprocessing: The input to a transformer-based video classification model is typically a sequence of video frames. These frames are usually preprocessed to have a consistent frame size, and the number of frames may be fixed or variable. Some common preprocessing steps include resizing, cropping, and normalizing the frames;
- Frame Embeddings: Each frame in the video is passed through a CNN or an optical flow network to extract visual features. These features are transformed into embeddings, which are then treated as the spatial information for each frame;
- Temporal Embeddings: In addition to spatial embeddings, a temporal embedding is created to capture the temporal relationships between frames. This can be achieved using techniques like positional encoding, which provides information about the relative positions of frames in the sequence;
- Input Encoding: Spatial and temporal embeddings are concatenated to create the input sequence. The Transformer model takes this sequence as input;
- Input Encoding:
  ○ Multi-Head Self-Attention: The self-attention mechanism allows the model to weigh the importance of each frame with others. Multiple attention heads are employed to capture different types of relationships;
  ○ Feed-forward Networks: After attention, a feed-forward neural network is applied to each frame’s representation;
  ○ Positional Encoding: The model incorporates positional encoding to differentiate frames within the sequence;
  ○ Layer Stacking: Multiple layers of self-attention and feed-forward networks are stacked to enable the model to learn complex spatiotemporal patterns;
- Pooling or Classification Head: After the transformer layers, a pooling operation can be applied to aggregate information across frames, or a classification head can be attached to make class predictions. This is where the spatial-temporal information is condensed into video-level features;
- Training: The model is trained on a labeled dataset of videos using a suitable loss function, such as categorical cross-entropy. Backpropagation and optimization techniques like Adam or RMSprop are used to update the model’s weights;
- Inference: Once the model is trained, it can be used for video classification by inputting video clips and obtaining class predictions based on the output of the pooling or classification head;
- Fine-tuning: Transfer learning from pre-trained models, such as those for image or text data, can be applied to improve performance. Fine-tuning video-specific datasets can adapt the model for video classification tasks.

### 3.6. Implementation Details

**Figure 12:**
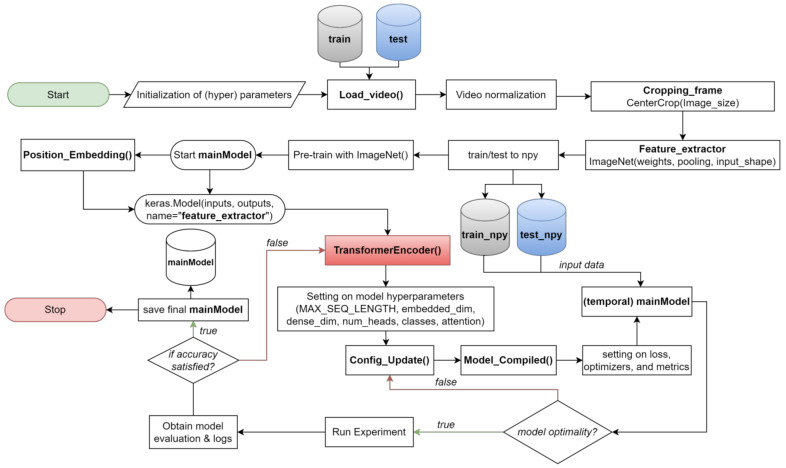
Process Flow of the Video Classification model using the Transformer model.

Our proposed model introduces an additional, fully connected layer for fine-tuning. We leverage pre-trained ImageNet models, including DenseNet121, ResNet50, VGG16, and VGG19, to benefit from their pre-trained weights and seamlessly append extra layers without jeopardizing the integrity of the pre-existing weights. We integrate positional encoding into the Transformer model to address the nuances of sequential videos. This process encompasses embedding frame positions through a dedicated layer and seamlessly integrating them with precomputed CNN feature maps, illustrated in Figure 12.

Our video classification model begins with importing the necessary libraries and initializing hyperparameters using Python’s TensorFlow and Keras libraries. Adjusting hyperparameters, including batch size, learning rate, and the number of epochs, are set to control the training process. We then load and preprocess video frames for feature extraction, ensuring consistent intensity levels through normalization and focusing on relevant information by cropping the center of each frame using the ‘Cropping_frame()’ function.

Next, we perform feature extraction using pre-trained ImageNet models (DenseNet121, ResNet50, VGG16, and VGG19) as feature extractors to transform each video frame into a compact representation capturing vital visual information. The ‘Feature_extractor’ function handles this process, and the resultant model is stored in a NumPy file, ensuring accessibility for future tasks.

With feature extraction complete, we define the main model, incorporating the Transformer architecture. Our transformer-based model comprises two custom layers: ‘PositionalEmbedding()’ and ‘TransformerEncoder()’.

The ‘PositionalEmbedding()’ layer is crucial for introducing positional information to the input sequence, aiding the Transformer model in understanding the temporal order of video frames. This temporal awareness improves video classification performance. The core of the Transformer model resides in the ‘TransformerEncoder()’ layer, which executes self-attention and feed-forward transformations. Implementing self-attention enables the model to selectively concentrate on various segments within the input sequence, prioritizing relevant frames at various time steps, while the feed-forward transformation captures intricate relationships and patterns between video frames.

The ‘TransformerEncoder()’ layer offers flexibility through various hyperparameters, including MAX_SEQ_LENGTH (controlling the maximum frames processed in a video sequence), embed_dim (determining feature representation dimensions), dense_dim (managing the intermediate dense layer units), and num_heads (indicating the number of attention heads). Higher attention head counts capture finer-grained relationships in video frames.

The ‘classes’ parameter defines the unique classes in the video classification dataset, influencing the output layer’s dimensionality and accurate class prediction. The ‘attention’ layer within ‘TransformerEncoder()’ implements the self-attention mechanism with multiple attention heads, enhancing the model by directing focus towards different segments of the video sequence during the training phase and contributing to better pattern learning.

For serialization and recreation of the ‘TransformerEncoder()’ layer, we implement the ‘Config_Update()’ method, providing a dictionary containing layer configuration. This enables saving and loading when serializing the model architecture custom properties like embed_dim, dense_dim, and num_heads. Once the model architecture is set, we compile the model using the ‘Model_Compiled()’ function, selecting sparse categorical cross-entropy as the loss function for video classification and employing the Adam optimizer for parameter optimization during training. Throughout training, key metrics such as accuracy, loss, and validation are monitored.

Following training, the model evaluates its generalization performance, which involves testing on an independent dataset to identify potential overfitting. As a final step, the trained model, including its weights and architecture, is saved for future use, allowing predictions on new videos without retraining.

## 4. Experiment, Results, and Discussion

In this section, we provide a comprehensive overview of our experimental approach, training procedure, and the results obtained from three distinct datasets: cloth freefall, cloth collision, and cloth aerodynamics. These datasets were generated through computer simulations utilizing the Position-Based Dynamics (PBD) method.

### 4.1. Experimental Setup

In our experiments, we meticulously organized the dataset into two separate directories: one for training and the other for testing. We allocated 880 videos for training and 400 videos for testing, distributed across five categories. We implemented our code using Python and TensorFlow with the Keras application, leveraging the CUDA library for accelerated training. The training process utilized the Adam optimizer, incorporating a learning rate set at 0.0001 and a training batch size of 32. We used a validation split of 0.16 for fine-tuning the models. The performance metric we utilized was the sparse categorical cross-entropy loss function, and all training was executed exclusively on the GPU. Detailed information regarding the hardware, software, parameters, and simulation settings is available in Table 5.

### 4.2. Results

In this section, we present the results of our experimental evaluations, providing a comprehensive analysis of the performance of different models in classifying cloth simulations. The subsequent section will delve into a more in-depth interpretation and discussion of these results.

#### 4.2.1. Performance Metrics on Different Datasets

Our evaluation process involved measuring precision, recall, accuracy, and F1-score for all categories within the video dataset, as shown in Table 6. The high values for these metrics indicate the effectiveness of the proposed models in classifying cloth simulations across various scenarios.

#### 4.2.2. Model Comparison and Analysis

To compare classification performance, we conducted experiments with four distinct models: RNN, GRU, LSTM, and Transformer. Each model underwent comprehensive pre-training using various architectures, including ImageNet, DenseNet121, ResNet50, VGG16, and VGG19. As demonstrated in Table 7, Table 8 and Table 9, the classification accuracy comparisons provide insights into the strengths and weaknesses of each model for different cloth simulation categories.

#### 4.2.3. Training Process and Robustness Evaluation

**Figure 13:**
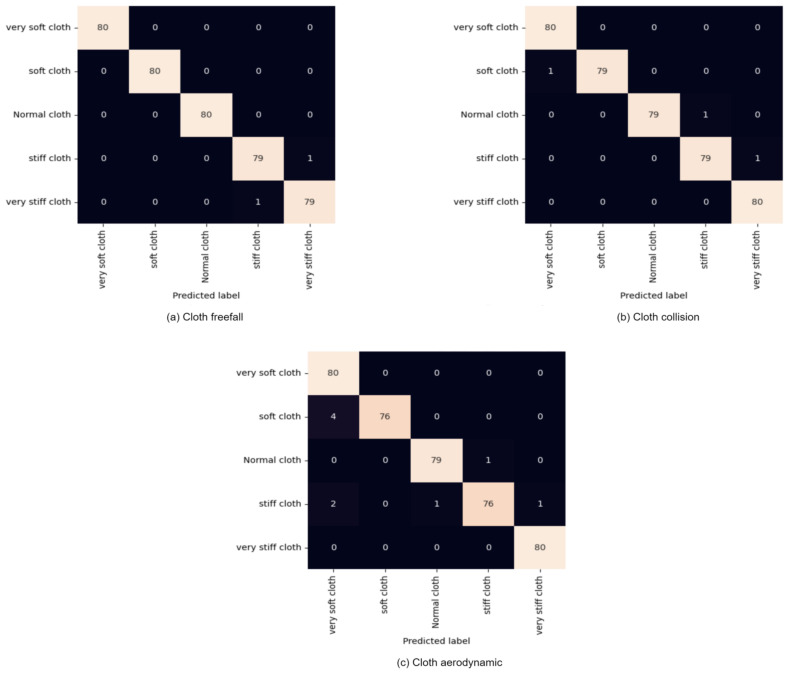
Confusion Matrix from our experiment datasets (**a**) cloth freefall, (**b**) cloth collision, and (**c**) cloth aerodynamics.

In the training and evaluation of our proposed methodology, the video dataset underwent batch processing, with 32 videos per batch fed to the training classifier. We configured the generator to apply various transformations, including random rotation and random shifting in both dimensions. Specifically, we introduced a random rotation of up to 70 degrees to the video frames and applied a random shift of 224 × 224 pixels to the input videos. The dataset was partitioned into 70% for training and 30% for testing, with an additional 16% allocated as a validation dataset within the training data. Through 250 epochs, the training process turned in a robust accuracy of 99.50%, as demonstrated in the confusion matrix in Figure 13a–c.

#### 4.2.4. Visual Representation and Training History

**Figure 14:**
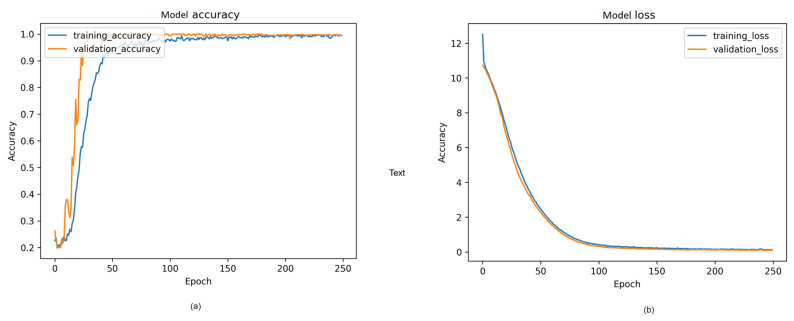
Proposed model training history from cloth freefall: (**a**) Model accuracy; (**b**) model loss.

**Figure 15:**
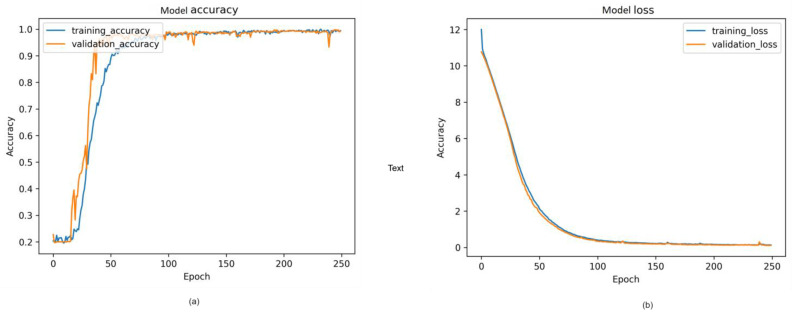
Proposed model training history from cloth collision: (**a**) Model accuracy; (**b**) model loss.

**Figure 16:**
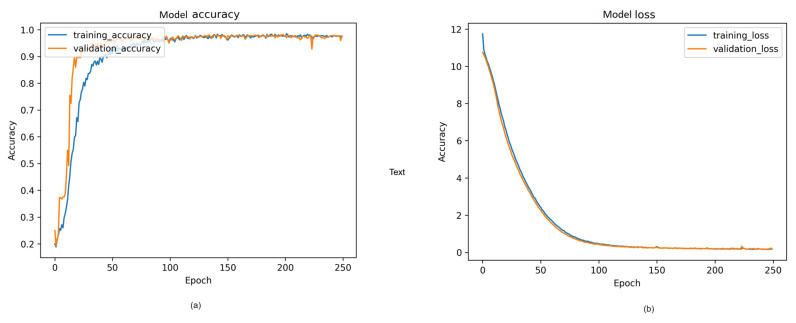
Proposed model training history from cloth aerodynamics: (**a**) Model accuracy; (**b**) model loss.

**Figure 17:**
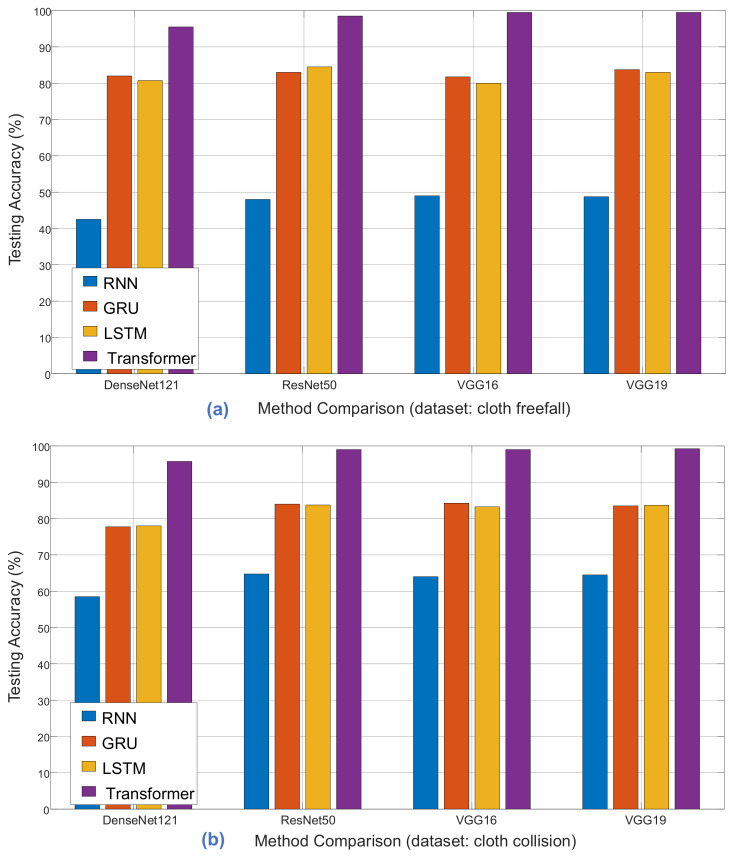

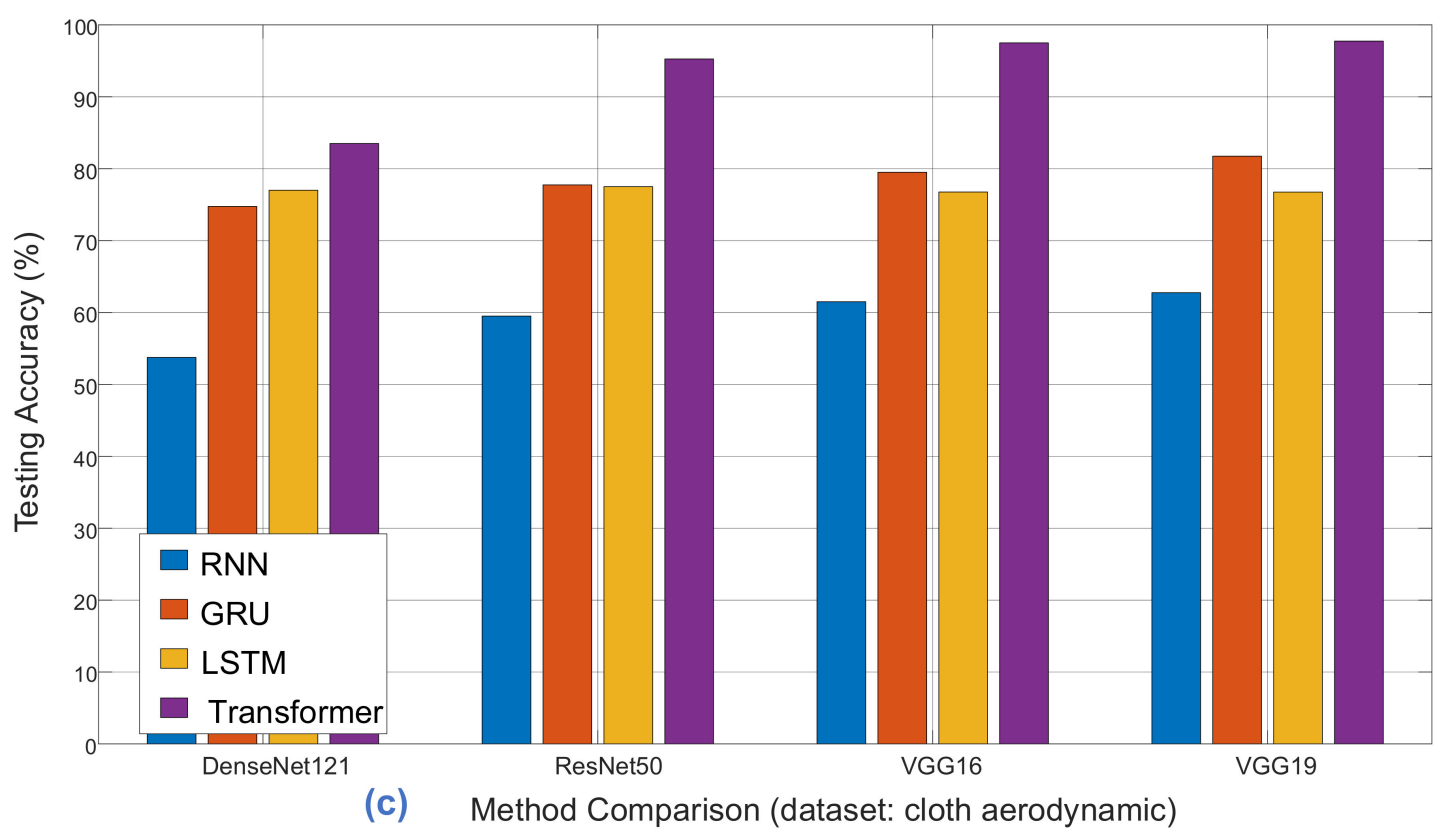
Illustrates the comparison of methods using three distinct categories of datasets: (**a**) cloth freefall, (**b**) cloth collision, and (**c**) cloth aerodynamics. We evaluated these datasets using various pre-trained models from the ImageNet model and four different main models. Our results show that all our proposed models are acceptable, but the pre-trained VGG19 and transformer models demonstrate the best accuracy and frame analysis performance.

Figure 14, Figure 15 and Figure 16 provide a visual representation of the model’s training history, offering insights into the convergence behavior and the learning process. The visualizations contribute to a better understanding of the training dynamics and the model’s performance over epochs. Additionally, in Figure 17, illustrate the comparison of models from three distinct categories of datasets. These datasets were evaluated using various pre-trained models from the ImageNet model and four different main models.

### 4.3. Discussion

In earlier investigations into video classification, scholars have documented a range of approaches grounded in traditional machine-learning methodologies through published press materials. These methods aim to identify multiple garments in real time, contributing significantly to the improvement of online virtual clothing style selection and, consequently, providing advantages to the broader clothing industry.

For instance, Medina et al. [29] introduced an approach that harnessed the capabilities of CNN and VGG16 for cloth classification, relying primarily on feature extraction. This method achieved an admirable accuracy rate of 90%. On the other hand, Chang et al. [30] harnessed region-based convolutional neural networks (R-CNN) in conjunction with YOLOv5s for feature extraction and fabric classification, culminating in a remarkable accuracy rate of 97%. It is worth noting, however, that both approaches confront inherent limitations stemming from the relatively modest size of their training datasets. This limitation resulted in a restricted number of images available for model training, impacting their capacity to generalize effectively. Additionally, practical challenges arose when certain clothing items ventured outside the field of view of the capturing camera, leading to inaccuracies in identifying specific cloth objects.

As shown in Table 10, the proposed approach outperformed alternative methods by combining VGG19 for feature extraction with the Transformer model. Our evaluation reveals that the deep learning model significantly enhanced accuracy in identifying and categorizing cloth simulations. This improvement was coupled with a noteworthy reduction in confusion time compared to other models. The observed efficiency can be credited to the parallel execution of the Transformer model, effectively preserving device resources throughout the execution process.

Our proposed model’s improved recognition and classification accuracy can be attributed to several key features. (i) The model demonstrates robustness to variations in physical properties, such as cloth color, brightness, random rotation angles, and image resolution in videos. (ii) The use of parallel processing for pre-trained model execution significantly reduces processing time compared to previous methods. (iii) The effective application of transfer learning techniques enables efficient training with substantial data. (iv) The model’s efficient execution during training leads to considerable time and cost savings. These advantages are achieved through a fully automated end-to-end architecture for feature extraction and classification, making our approach a compelling solution for various video analysis tasks.

## 5. Conclusions

In this paper, we propose a model for cloth video scene classification, focusing specifically on cloth video types. To evaluate our model’s efficacy, we systematically assessed its performance, comparing it to the state-of-the-art model and recent research in the same domain. Our evaluation utilized cloth videos created through the PBD method for the cloth simulation dataset, classifying them into five distinct categories: very soft cloth, soft cloth, normal cloth, stiff cloth, and very stiff cloth. To enhance the accuracy of video classification, we integrated pre-trained ImageNet models, including DenseNet121, ResNet50, VGG16, and VGG19, with a Transformer model. Our experiments were conducted on the PBD cloth simulation dataset, which comprises three separate clothing datasets: cloth freefall, cloth collision, and cloth aerodynamic. Each of these datasets encompasses five categories for predicting clothing attributes. Furthermore, we introduced a parallel processing technique on the pre-trained step to read frames from the video dataset efficiently, resulting in significant time savings.

Our results clearly illustrate that the combination of VGG19 and the Transformer model surpasses other architectural configurations, achieving an outstanding accuracy rate of 99.50%. Our experiments covered three distinct dataset types, encompassing 3840 videos. Our proposed methodology was primarily evaluated on cloth video data as a foundational element for video summarization; its versatility extends to a wide range of applications, including image analysis, movie recommendations, biological organism classification, self-driving vehicles, and other domains where precision and low error rates are of paramount importance. Compared to previously proposed ones, our model’s exceptional performance positions it as a robust candidate for reuse in diverse applications. Scene classification can be further expanded into research on video description, medical video analysis, and event description, while cloth video analysis remains a prominent and evolving field, demanding continuous efforts to enhance task quality.

For future work, we also plan to investigate the potential of our model for more complex datasets from simulation and datasets from real-world problems. While our model was evaluated on a simulated dataset, how well it performs on real-world cloth video scenes captured by cameras remains to be seen. Therefore, future work could involve collecting and annotating real-world cloth video datasets and evaluating our model on these datasets. This could involve addressing additional challenges, such as camera motion, lighting variations, and occlusions that are not present in the simulated dataset.

## Figures and Tables

**Table 1:** Pre-trained models for reading frames in video classification.

Pre-Trained Models	Arguments	Number of Features
DenseNet121	(128, 128, 3)	1024
ResNet50	(224, 224, 3)	2048
VGG16	(224, 224, 3)	512
VGG19	(224, 224, 3)	512

**Table 2:** Computation time in seconds (s) for comparing video parallel frame readings.

Processors	Folders	DenseNet121	ResNet50	VGG16	VGG19
CPU	train	2576.64 s	2701.93 s	2823.16 s	3334.86 s
GPU	train	445.78 s	491.95 s	364.00 s	406.55 s
CPU	test	850.99 s	1119.15 s	1259.33 s	1461.54 s
GPU	test	233.36 s	175.92 s	137.29 s	142.021 s

**Table 3:** Parameters used to generate the cloth dataset.

Duration	Mass	Damping	Dt	Iteration	Stiffness
30 s	10 20 30 40	0.001 0.0025 0.0075 0.01	0.01 0.02 0.04 0.05	5 20 40 50	0.1 0.25 0.50 0.75 1.0

**Table 4:** Extract the video names and labels from the PBD dataset.

Video Name	Labels	Types	Length
PBD_Freefall_stf0.1_c0100.avi	stf0.1	.avi	00:00:30
PBD_Freefall_stf0.1_c0101.avi	stf0.1	.avi	00:00:30
PBD_Freefall_stf0.25_c0102.avi	stf0.25	.avi	00:00:30
PBD_Freefall_stf0.25_c0103.avi	stf0.25	.avi	00:00:30
PBD_Freefall_stf0.50_c0104.avi	stf0.50	.avi	00:00:30
PBD_Freefall_stf0.50_c0105.avi	stf0.50	.avi	00:00:30
PBD_Freefall_stf0.75_c0106.avi	stf0.75	.avi	00:00:30
PBD_Freefall_stf0.75_c0107.avi	stf0.75	.avi	00:00:30
PBD_Freefall_stf1.0_c0108.avi	stf1.0	.avi	00:00:30
PBD_Freefall_stf1.0_c0109.avi	stf1.0	.avi	00:00:30

**Table 5:** Software and simulation parameters.

Software/Parameters	Value
Windows 10	64-bit
Programming Language	Python, Keras, TensorFlow
CPU	AMD Ryzen 9 5900x
GPU	NVIDIA GeForce RTX 4090
RAM	128 GB
Batch size	32
Validation split	0.16
Optimizer	Adam
Learning rate	0.0001
Loss function	sparse categorical cross-entropy
Epochs	250
Dataset	3840 videos

**Table 6:** Comparison of datasets for experiments on classification performance.

Dataset Type	Accuracy	Precision	Recall	F1-Score
Cloth freefall	0.99	1.00	0.99	0.99
Cloth collision	0.99	0.99	0.99	0.99
Cloth aerodynamic	0.98	0.98	0.98	0.98

**Table 7:** Classification accuracy comparison with RNN, GRU, LSTM, and Transformer models (cloth freefall).

Method	RNN	GRU	LSTM	Transformer
DenseNet121	42.50	82.00	80.75	95.50
ResNet50	48.00	83.00	84.50	98.50
VGG16	49.00	81.75	80.00	99.50
VGG19	48.75	83.75	83.00	99.50

**Table 8:** Classification accuracy comparison with RNN, GRU, LSTM, and Transformer models (cloth collision).

Method	RNN	GRU	LSTM	Transformer
DenseNet121	58.50	77.75	78.00	95.75
ResNet50	64.75	84.00	83.75	99.00
VGG16	64.00	84.25	83.24	99.00
VGG19	64.50	83.50	83.65	99.25

**Table 9:** Classification accuracy comparison with RNN, GRU, LSTM, and Transformer models (cloth aerodynamic).

Method	RNN	GRU	LSTM	Transformer
DenseNet121	53.75	74.75	77.00	83.50
ResNet50	59.50	77.75	77.50	95.25
VGG16	61.50	79.50	76.75	97.50
VGG19	62.75	81.75	76.75	97.75

**Table 10:** Model comparison table with state-of-the-art models.

Authors	Methods		Accuracy
	Feature Extraction	Classification On	
Medina et al. [29]	CNN + VGG16	Cloth	0.90
Chang et al. [30]	R-CNN + YOLOv5s	Fabric	0.97
The proposed method	Transformer + VGG19	Cloth	0.99

## Data Availability

No new data were created or analyzed in this study. Data sharing is not applicable to this article.
